# Study of diatom assemblages in Sundarbans mangrove water based on light microscopy and *rbc*L gene sequencing

**DOI:** 10.1016/j.heliyon.2018.e00663

**Published:** 2018-06-27

**Authors:** Brajogopal Samanta, Punyasloke Bhadury

**Affiliations:** Integrative Taxonomy and Microbial Ecology Research Group, Department of Biological Sciences, Indian Institute of Science Education and Research Kolkata (IISERK), Mohanpur 741246, Nadia, West Bengal, India

**Keywords:** Microbiology, Ecology, Environmental sciences

## Abstract

Sundarbans, the world's largest mangrove deltaic region, is one of the most productive ecosystems in tropical and subtropical latitudes and also serve as a nursery ground for rich coastal fisheries. In this study, we highlighted diatom assemblages from the Indian part of Sundarbans Biosphere Reserve (SBR) area for the first time based on light microscopy and *rbc*L gene sequencing and phylogeny. In total, 15 diatom species (11 centric forms and 4 pennate forms) were documented using light microscopy, and 3 major clades of diatoms were detected in *rbc*L phylogeny. Out of 15 diatom species, 7 were the first record from Sundarbans mangrove water. One of the species, *Thalassiosira ferelineata* Hasle and Fryxell, was reported for the first time in an Asian mangrove ecosystem based on light microscopy. Our study suggests the importance of establishing cultures and their polyphasic taxonomy are the future necessity to create an authenticated diatom database from mangrove water, which is still overlooked globally.

## 1 Introduction

Marine ecosystems are key contributors of global primary production accounting for approximately 50 gigatons of carbon each year (Field et al., 1998). One of such productive ecosystems is mangrove ecosystem located in tropical and subtropical latitudes. This ecosystem also serves as the nursery ground for rich fisheries (Robertson and Blaber, 1992). The sustenance of fishery resources in mangrove ecosystem is mainly due to plankton communities including phytoplankton (Robertson and Blaber, 1992). Sundarbans, the world's largest deltaic contiguous mangrove region, is situated at the estuarine phases of river Ganga, Brahmaputra and Meghna between latitudes 21°32′ and 21°40′ N and longitudes 88°05′ and 89° E spanning across India and Bangladesh (Spalding et al., 1997). The salient features of this deltaic mangrove ecosystem include interconnected network of numerous creeks, rivers and rivulets, and semi-diurnal tidal influences across the entire region. In spite of floristic diversity of mangroves (Barik and Chowdhury, 2014), the large variability in hydrological parameters including both freshwater and coastal water influences, topographic heterogeneity and their interactions, has resulted in rich biodiversity in Sundarbans (Gopal and Chauhan, 2006). In 1987, UNESCO declared the core mangrove forest of Indian Sundarbans (2,585 sq. km) as Sundarbans Biosphere Reserve (SBR) for protection and conservation of mangrove flora and fauna including the Royal Bengal Tiger from anthropogenic disturbances.

Phytoplankton, in particular chromophytic phytoplankton, is the major contributor to aquatic primary production in Sundarbans mangrove (e.g., Biswas et al., 2010; Manna et al., 2010; Bhattacharjee et al., 2013; Samanta and Bhadury, 2014). Phytoplankton community fingerprinting techniques based on molecular tools (e.g. gene sequencing and phylogeny) along with microscopy has been found to be promising approaches for accelerating biodiversity research in Sundarbans mangrove ecosystem (Bhattacharjee et al., 2013; Samanta and Bhadury, 2014, 2016). The high fish productivity in Sundarbans also implicitly depends on net primary production by phytoplankton (Mukherjee et al., 2013). Diatoms have been found to overwhelmingly dominate phytoplankton assemblages (>75%) in this mangrove ecosystem (Biswas et al., 2010; Manna et al., 2010; Aziz et al., 2012; Samanta and Bhadury, 2014). Diatoms form the basis of world's shortest and most efficient food webs such as sustenance of rich fisheries in coastal ecosystems including mangroves (e.g., Jickells, 1998). They are used as a biological proxy to monitor health of coastal ecosystems (e.g., Stevenson et al., 2008; Vilmi et al., 2015); however studies are limited in this aspect with respect to mangroves (Smol and Stoermer, 2010). Therefore, it is necessary to establish baseline information of diatom assemblages from mangroves to test their potential as a reliable bioindicator for assessment of environmental health and also for fisheries stock assessment such as planktivorous fishes. The information of diatom assemblages albeit in limited numbers is available from different parts of Sundarbans mangrove ecosystem; however the Indian part of Sundarbans Biosphere Reserve (SBR) region has remained largely unexplored. The SBR which is a protected zone, a pristine environment (lesser human interference) represent a unique opportunity to monitor natural diatom assemblages and their importance in this area of Sundarbans mangrove ecosystem. In the present study, we documented novel insights into diatom assemblages from the Indian part of SBR area based on light microscopy and *rbc*L phylogeny to establish the baseline information for future diatom based ecosystem health monitoring of this ecosystem.

## 2 Methods

### 2.1 Study area and sample collections

The Indian part of SBR is situated in the eastern part of Indian Sundarbans adjoining Bangladesh and bound by Matla River in the west and southern part facing the Bay of Bengal. The study area is strongly influenced by riverine freshwater as well as saline water from Bay of Bengal. Consequently, a salinity gradient is formed across the study sites. The area can be divided into three inter-related zones: core area which is largely protected from any external disturbance, buffer zone, and Sajnekhali Sanctuary. Surface water was collected (50 mL) for undertaking phytoplankton analysis from five different stations of SBR (see Fig. 1) during the month of November and December 2012 at low tide following standard protocol (Bhattacharjee et al., 2013). The selection of stations were undertaken based on prevailing salinity gradient with stations closer to the coastal Bay of Bengal had higher salinity than stations away from the Bay of Bengal. Moreover, the stations which are largely shallow located in the area of SBR which has rich mangrove species and also serve as the nursery ground of planktivore fish populations. Following collection, samples were immediately fixed with molecular grade 100% ethanol (Merck, Billerica, MA, USA) with a ratio of 1:5 (v/v) ethanol: sample. At the time of sampling, salinity was measured *in situ* using a refractometer (Erma, Japan) in each station (Table 1). The stations StnSBRS1, S2 and S3 were located in core area; StnSBRN1 in buffer zone; and StnSBRN2 in Sajnekhali Sanctuary of SBR (Fig. 1).

**Fig. 1:**
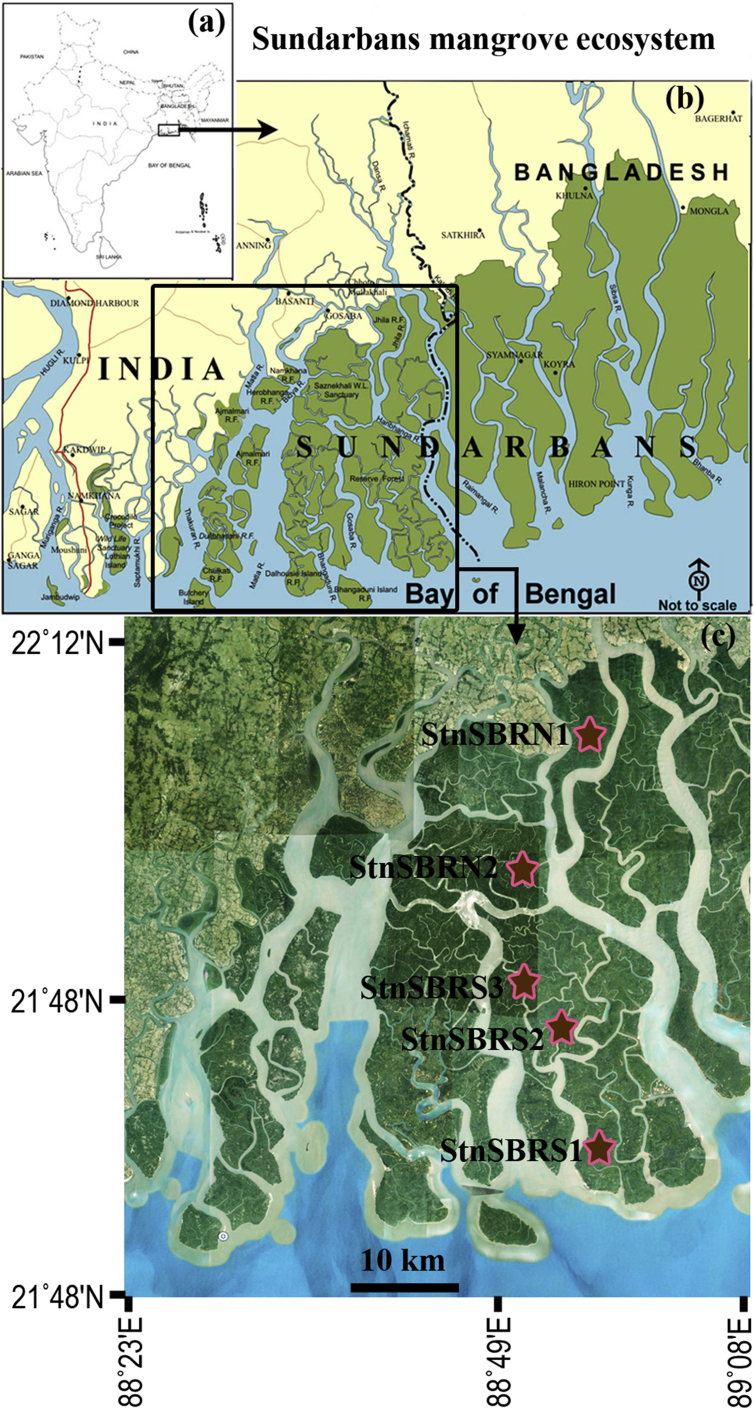
Maps of the study area. (a) Country map of India and Bangladesh. (b) The entire Sundarbans mangrove ecosystem across India and Bangladesh. (c) The map showing the stations sampled in Indian part of SBR; StnSBRN1 in buffer zone, StnSBRN2 in Sajnekhali Sanctuary and StnSBRS1, S2, and S3 in core area of SBR. Coordinate of stations sampled are mentioned in Table 1 along with *in situ* salinity values.

**Table 1:** List of diatoms species recorded using light microscopy from five stations of SBR. Taxa marked as bold indicate first report from Indian part of Sundarbans. Salinity and the number of *rbc*L clones that were sequenced from each sampling station are also mentioned.

Sampling station	Coordinates	Salinity (psu)	Diatom species encountered in light microscopy	Numbers of *rbc*L clones sequenced
StnSBRN1	22°05′51.70″N 88°57′19.30″E	15	***Coscinodiscus argus****, Cyclotella striata,****C. litoralis, Ephemera planamembraneacea****, Entomoneis alata,****Shionodiscus oestrupii* var. *venrickae, T. eccentrica****, Thalassiosira* sp.	8
StnSBRN2	21°56′50.90″N 88°52′37.90″E	18	*Bacillaria* sp., ***Coscinodiscus argus****, C. marginatus, Cyclotella striata,****C. litoralis, Ephemera planamembraneacea****, Lauderia annulata,****Thalassiosira eccentrica****, Thalassiosira* sp.	9
StnSBRS1	21°40′28.87″N 88°57′28.58″E	15	*Coscinodiscus marginatus*, ***Cyclotella litoralis, Thalassiosira ferelineata, T. eccentrica, T. nodulolineata***	25
StnSBRS2	21°47′35.27″N 88°55′19.11″E	19	***Coscinodiscus argus****, C. jonesianus, Cyclotella striata,****C. litoralis****, Thalassiosira* sp.	12
StnSBRS3	21°49′45.37″N 88°53′00.42″E	20	*Bacillaria* sp., ***Coscinodiscus argus****, Cyclotella striata, Surirella* sp., ***Thalassiosira eccentrica****, Thalassiosira* sp.	13

### 2.2 Microscopic observations and molecular analyses

For diatom cell count, 25 ml of preserved water samples were concentrated by gravity sedimentation (24 h) and subsequently enumerated based on drop count method under a light microscope BX53 (Olympus, Tokyo, Japan) (Verlencar and Desai, 2004). Water mount samples were observed for species level identification of diatoms under a light microscope BX53 (Olympus), and differential interference contrast (DIC) images were captured using CellSens software (Olympus).

Environmental DNA was extracted from 25 mL of water sample from each station using a modified DNA extraction protocol of Boström et al. (2004) with respect to two steps. In one step, 5 μL of proteinase K (10 mg mL^−1^) (Amresco, USA) was added during lysis and incubated at 55 °C for 3 hours. In the next step, 10 μL of lysozyme (20 mg mL^−1^) (SRL, India) was added and incubated at 37 °C for 1 hour. Rest of the extraction protocol was same as detailed earlier by Boström et al. (2004). Partial *rbc*L gene fragments (554 bp) were amplified from environmental DNA for all the stations using *rbc*L primers (Wawrik et al., 2002; Samanta and Bhadury, 2014). PCR reactions were performed in triplicate for each environmental DNA sample, pooled together and subsequently purified using Gel Purification Kit (Qiagen, Valencia, USA) as per manufacturer's protocol. Purified PCR products were cloned using pGEM-T Easy vector (Promega, Madison, USA) following manufacturer's instructions. Sequencing was undertaken with SP6 primer in ABI Prism 3130 Genetic Analyzer based on BigDye Terminator Chemistry (Foster City, CA, USA) following manufacturer's instructions.

The base calling of chromatograms were manually checked and vector sequences were trimmed in the two ends of each *rbc*L sequences in BioEdit v7.0 (Hall, 1999) before undertaking further analysis. The generated DNA sequences were compared with published *rbc*L sequences using blastn tool (http://blast.ncbi.nlm.nih.gov/Blast.cgi). Two hundred fifty *rbc*L reference sequences from databases with the sequences generated in this study were aligned using MUSCLE (http://www.ebi.ac.uk/Tools/msa/muscle/). Phylogenetic analyses were undertaken at the nucleotide level. Bayesian inference (BI) was performed using MrBayes v3.2 by selecting GTR+I+G substitution model (Ronquist et al., 2012). A four chains (two cold and two heated) run for 100 millions generations was used and trees were sampled every 5000 generation. Posterior probabilities (PP) were estimated with 50% burn-in, and majority rule consensus tree was constructed. An online version of PHYML (http://www.atgc-montpellier.fr/phyml/; Guindon et al., 2010) was used to construct a maximum-likelihood (ML) tree by selecting AIC criterion for substitution model selection (Lefort et al., 2017). The *rbc*L sequences generated as part of this study have been submitted to GenBank and their accession numbers are from KT335295-304, KT335317-324, and KT335346-398.

## 3 Results and discussion

**Fig. 2:**
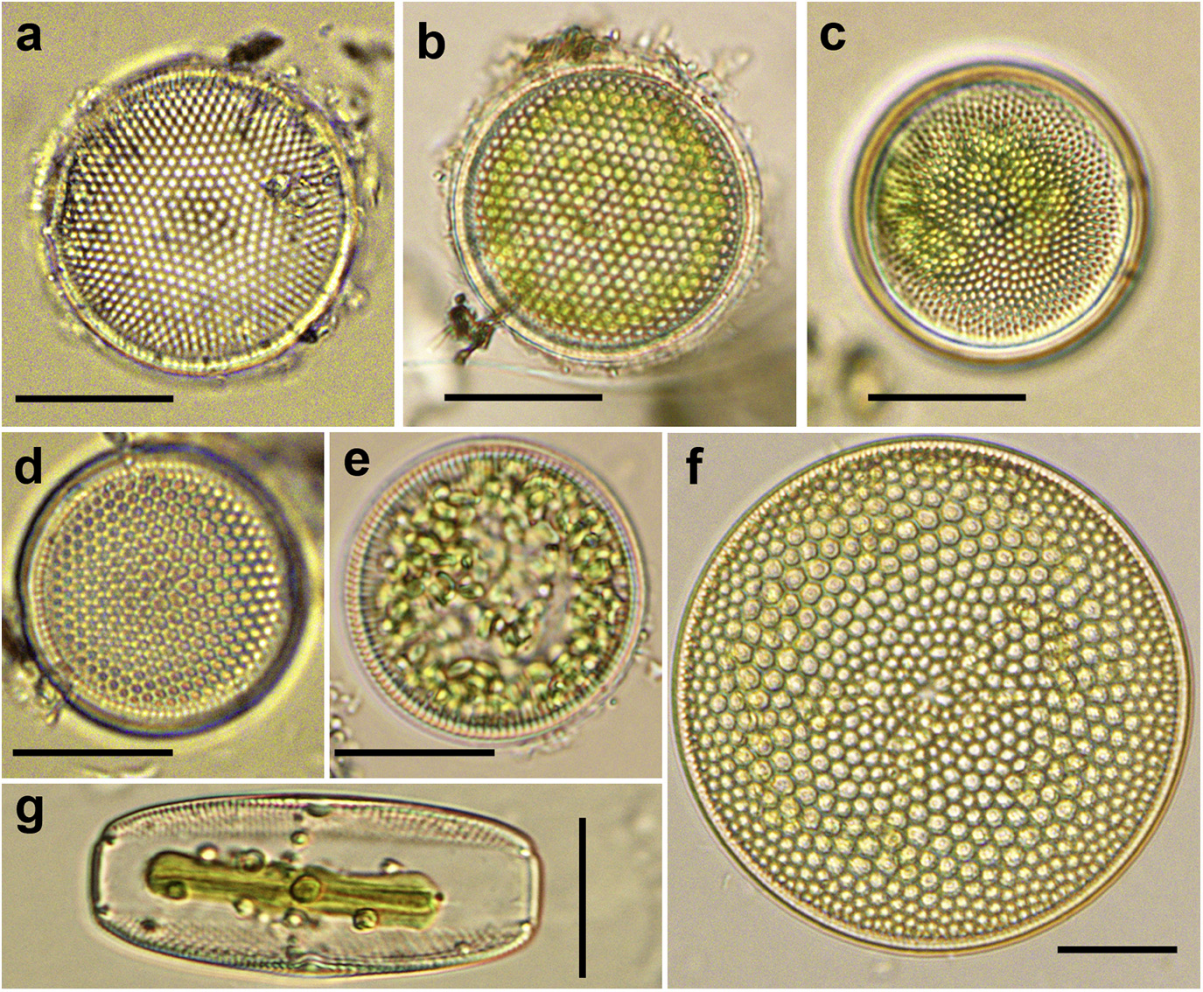
Differential interference contrast (DIC) images of diatom species reported for the first time from Indian Sundarbans. (a) *Thalassiosira eccentrica.* (b) *T. ferelineata.* (c) *Shionodiscus oestrupii* var. *venrickae.* (d) *T. nodulolineata.* (e) *Cyclotella litoralis.* (f) *Coscinodiscus argus.* (g) *Ephemera planamembranacea.* Scale bars = 20 μm.

**Fig. 3:**
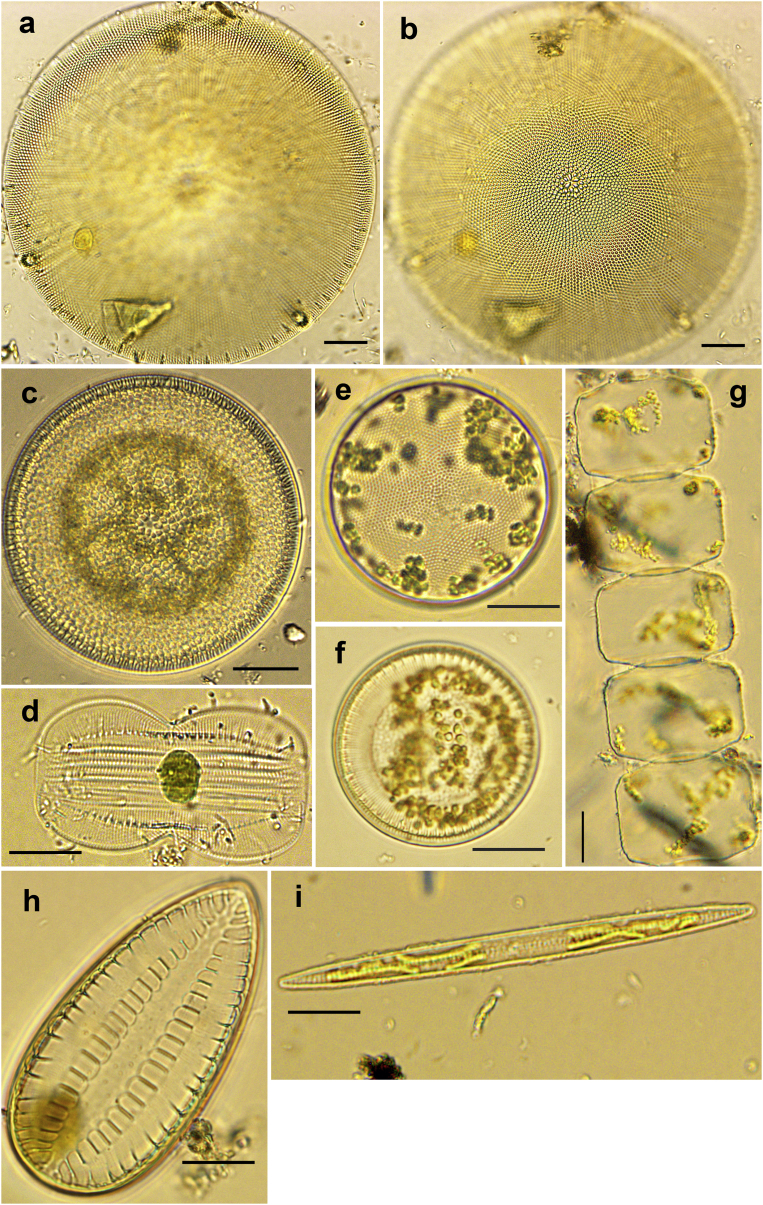
Differential interference contrast (DIC) images of diatoms encountered from SBR using light microscopy. (a–b) *Coscinodiscus jonesianus.* (c) *C. marginatus.* (d) *Entomoneis alata.* (e) *Thalassiosira* sp. (f) *Cyclotella striata.* (g) *Lauderia annulata.* (h) *Surirella* sp. (i) *Bacillaria* sp. Scale bars = 20 μm.

Diatom cell abundance at our study area ranged from 12,500 cells/L (StnSBRS1) to 23,000 cells/L (StnSBRN2) (Table 1). In Sundarbans mangrove water, phytoplankton cell density can vary from 1,000 s (Bhattacharjee et al., 2013) to 100,000 s of cells/L (Choudhury et al., 2015). It is anticipated that diatom cell abundance in SBR could be similar to other part of this ecosystem studied so far. A total of 15 diatom species were identified across five stations in the Indian part of SBR based on light microscopy (Figs. 2a–g and 3). The number of diatom species per station ranged from 4 (StnSBRS1) to 9 (StnSBRN2). Stations with higher salinity also had a higher number of diatom species and cell abundance (Table 1). Out of the 15 identified diatom species, only 4 were pennate forms, while the rest were centric forms. The pennate diatom species were only encountered in StnSBRN1 (2 species), StnSBRN2 (1 species) and StnSBRS3 (2 species) (Table 1). Our result showed relatively low diatom species diversity in SBR compared to other parts of the ecosystem (e.g. Biswas et al., 2010; Manna et al., 2010; Bhattacharjee et al., 2013). Moreover, the study area is represented by shallow water depth and very high suspended particulate matter load which limits light penetration and resulting low abundance of phytoplankton cells such as diatoms as found in this ecosystem (e.g. Bhattacharjee et al., 2013). Also, limited sampling effort due to accessibility issues and logistic could be one of the reasons in addition to possible role of temporal variability in diatom populations that may have accounted for observed low diatom species diversity. The described diatom diversity is a small fraction of total, as only 50 ml of sample was collected to study the diversity. For phytoplankton monitoring usually large volume of water is sieved through plankton net. It is expected that if the sampling effort is increased seasonally then more diatom species could be detected from the study area. However, it is important to mention that our study area is largely restricted from human intrusion.

Out of 15 encountered diatom species solely based on light microscopic identification, six species namely, *Thalassiosira ferelineata* Hasle and Fryxell, *Thalassiosira nodulolineata* (Hendey) Hasle and Fryxell, *Shionodiscus oestrupii* var. *venrickae* (Fryxell and Hasle) Alversion, Kang and Theriot, *Coscinodiscus argus* Ehrenberg, *Cyclotella litoralis* Lange and Syvertsen, and *Ephemera planamembranacea* (Hendey) Paddock were recorded for the first time from Sundarbans. *Thalassiosira eccentrica* (Ehrenberg) Cleve was recorded for the first time from Indian part of SBR. Illustrated accounts including taxonomy and distribution of these species including some as new records from this ecoregion and also from Asia have been provided below.

*Thalassiosira eccentrica* (Ehrenberg) Cleve (Fig. 2a)

*Basionym: Coscinodiscus eccentricus* Ehrenberg

*References:* Fryxell and Hasle, 1972, p. 300, Figs 1–18; Harris et al., 1995, p. 123, Fig. 29; Hasle and Syvertsen, 1996, p. 62; Park et al., 2012, Fig. 5U; Li et al., 2013, p. 91–93, Fig. 47.

*Descriptions:* Valves faces are flat and cell diameter varies from 40–50 μm. Areolae are in curved tangential rows (eccentric structure) with a tendency towards fasciculation. Five to eight areolae are present in 10 μm. In addition to the eccentric areolae structure on the valve face, the central areola is surrounded by seven areolae is the characteristic diagnostic feature of *T. eccentrica* under light microscope. *T. angulata* (Gregory) Hasle and *T. pacifica* Gran and Angst also have the eccentric areolae patterns, but they have absence of central areola is surrounded by seven areolae.

*Distribution:* This species is cosmopolitan, usually found in Pacific, Atlantic, and Indian Oceans, more specifically, in Japan as well as in East and South China Sea regions (Li et al., 2013). Previously this species was reported from Rupsha, Passur, Khalpatura, Arpangachia, Bhola, and Baleswar river systems of Bangladesh part of Sundarbans (Rahaman et al., 2013), but this is the first report from Indian part of Sundarbans.

*Thalassiosira ferelineata* Hasle and Fryxell (Fig. 2b)

*References:* Hasle and Fryxell, 1977, P. 26–28, Figs 46–53; Hasle and Syvertsen, 1996, p. 59; Lehmkuhl et al., 2010, p. 316, Fig. 6.

*Descriptions:* Valve face is flat with narrow mantle flaring to broad marginal rim, 39–41 μm in diameter. Hexagonal areolae are in straight tangential rows with six to seven areolae in 10 μm. Additional row of small areolae are present at the valve margin. Diameter of marginal areolae are less than half that at the center. *T. tenera* Hasle and Fryxell also has the similar areolae pattern as *T. ferelineata* in light microscopic observation. These two species can be distinguished by their valve diameter. The valve diameter of *T. ferelineata* is higher than *T. tenera*. The valve diameter of *T. tenera* generally varied from 10–29 μm.

*Distribution:* This species is mainly found in warm water regions (Hasle and Syvertsen, 1996) and also reported from Antarctic and sub-Antarctic islands (Scott and Thomas, 2005). This is the first report of this species not only from Sundarbans mangrove, but also from Asia.

*Shionodiscus oestrupii* var. *venrickae* (Fryxell and Hasle) Alversion, Kang and Theriot (Fig. 2c)

*Basionym: Thalassiosira oestrupii* var. *venrickae* Fryxell and Hasle

*Reference:* Fryxell and Hasle, 1980, p. 810–813, Figs 11–19

*Descriptions:* Cells are 40 μm in diameter. Six to twelve areolae are present in 10 μm on valve face and areolae usually larger in central part of valve than closer to the margin. Areolae patterns are radial to sub-linear array.

*Distribution:* This species is generally found in warm water to temperate regions (Fryxell and Hasle, 1980). It has been previously reported as *Thalassiosira oestrupii* from Rupsha, Passur, Bhola and Baleswar river systems of Bangladesh part of Sundarbans (Rahaman et al., 2013). This is the first report from Indian part of Sundarbans.

*Thalassiosira nodulolineata* (Hendey) Hasle and Fryxell (Fig. 2d)

*Basionym: Coscinodiscus nodulolineatus* Hendey

*References:* Hasle and Fryxell, 1977, p. 35, Figs 86–93; Torgan and Santos, 2007, p. 716, Figs 1A–C; Lehmkuhl et al., 2010, p. 316, Fig. 10; Li et al., 2013, p. 99, Figs 95–99.

*Descriptions:* Cells are 28–30 μm in diameter. Hexagonal areolae arranged in straight tangential rows with six to twelve areolae are present in 10 μm on valve face. Central areola is slightly larger. The valve mantle is characteristically ornamented with short ribs. *T. nodulolineata* can be easily confused with *T. nanolineata* (Mann) Fryxell in light microscopy. *T. nanolineata* has strutted process close to the central areolae, while in *T. nodulolineata* has the strutted process inside the central areolae which is visible based on light microscopy (Torgan and Santos, 2007).

*Distribution:* This taxon has a cosmopolitan distribution in coastal water. It has been recorded frequently reported from South China Sea (Li et al., 2013). However, this is the first report from Sundarbans mangrove water.

*Cyclotella litoralis* Lange and Syvertsen (Fig. 2e)

*Descriptions:* Cell diameter varies from 25–30 μm with undulated valve face. Ten to eleven striae are present in 10 μm. Marginal strutted processes are present on every interstria. *C. litoralis* differs from *C. striata* by the fact that in the later species undulation of the central area of the valve does not reach the region of marginal ridges.

*References:* Lange and Syvertsen, 1989, p. 343–344, Figs 1–30; Park et al., 2012, Fig. 5i; Lee et al., 2015, p. 587, Figs 8c–d.

*Distribution:* This taxon is usually found in marine aquatic ecosystem encompassing Northern and Southern temperate regions (Hasle and Syvertsen, 1996). The presence of this taxon is the first report from Sundarbans mangrove ecosystem.

*Coscinodiscus argus* Ehrenberg (Fig. 2f)

*References:* Hasle and Sims, 1986, p. 33–34, Figs 1–7; Hasle and Syvertsen, 1996, p. 103.

*Descriptions:* Cells are 75–85 μm in diameter. Small central hyaline area is present. Areolae pattern is radial and hexagonal in shape. Areolae increase in size from center of the valve towards the middle of radius, then decrease in size towards the margin. Two to six areolae is present in 10 μm on valve surface. *Coscinodiscus radiatus* Ehrenberg also have radial areolae pattern, however, *C. argus* can be distinguished from *C. radiatus* by areolae of uniform size throughout the entire valve face in the later species under light microscope.

*Distribution:* This species has a cosmopolitan distribution (Hasle and Syvertsen, 1996), but in this study, for the first, reported from Sundarbans.

*Ephemera planamembranacea* (Hendey) Paddock (Fig. 2g)

*Basionym: Navicula planamembranacea* Hendey

*References:* Hendey, 1964, p. 188, Fig. 8; Paddock, 1988, p. 86, Plate 31; Hasle and Syvertsen, 1996, p. 284, Plate 64.

*Descriptions:* Cells usually solitary. Apical axis, 66–69 μm; Parvalver axis, 29–30 μm. Central nodules are slightly depressed. Due to the shape of the cell this species it is usually seen in girdle view under the light microscope. In the girdle view, central and terminal nodules of the raphae are distinct. Small raphae fins are present near valve end. Hasle and Syvertsen (1996) considered that *E. planamembranacea* is more similar to *Tropidoneis* than to *Navicula sensu stricto* although, in the past, it has never been referred to *Tropidoneis*.

*Distribution:* This genus is mainly found in North Atlantic Ocean (Hasle and Syvertsen, 1996) and also reported from Arctic and Antarctic regions (Scott and Thomas, 2005; Mather et al., 2010). This species is the first report from Sundarbans.

**Figure S2:**
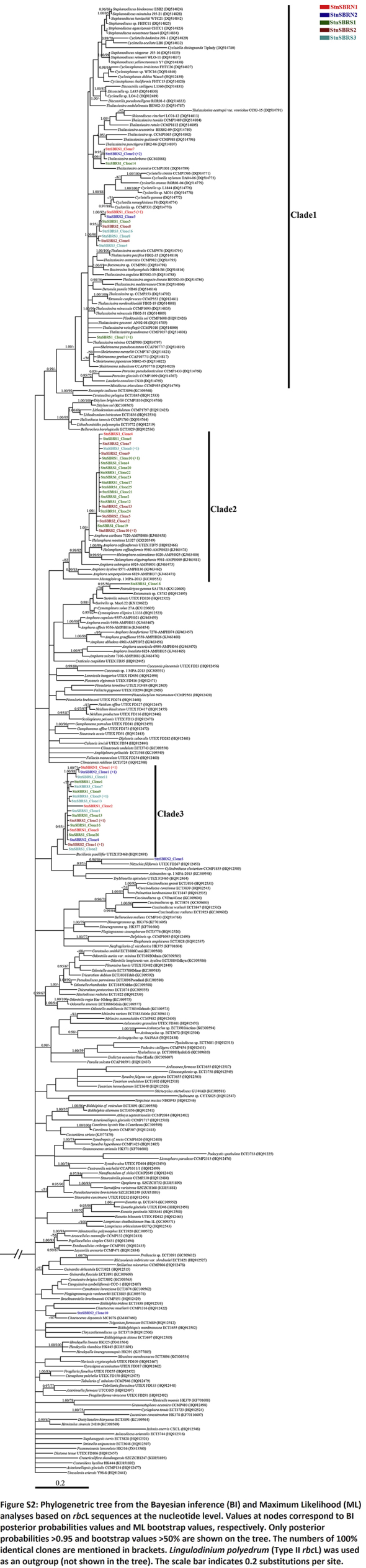
Phylogenetic tree from the Bayesian inference (BI) and Maximum Likelihood (ML) analyses based on *rbc*L sequences at the nucleotide level. Values at nodes correspond to BI posterior probabilities values and ML bootstrap values, respectively. Only posterior probabilities >0.95 and bootstrap values >50% are shown on the tree. The numbers of 100% identical clones are mentioned in brackets. *Lingulodinium polyedrum* (Type II *rbc*L) was used as an outgroup (not shown in the tree). The scale bar indicates 0.2 substitutions per site.

Additionally, *rbc*L gene sequencing from environmental DNA samples provided valuable information on diatom assemblages at the phylogenetic level from this ecologically important mangrove ecosystem facing the coastal Bay of Bengal. A total of 67 *rbc*L clones were sequenced from five different stations of SBR (Table 1). The basal topology of the *rbc*L tree is polytomy (Fig. S2). In *rbc*L phylogenetic tree, majority of the environmental *rbc*L clones were grouped into three major clades (Fig. S1; Fig. S2). In clade1, a total of 16 *rbc*L clones clustered with species of thalassiosiroid diatoms (Fig. S1). Among these 16 *rbc*L clones, nine clones formed a separate sub-clade within the clade1. This sub-clade did not include any culture thalassiosiroid diatom *rbc*L sequences and also had strong statistical support both in ML (bootstrap value 90%) and Bayesian (posterior probability value 1.00) analyses. This sub-clade may be representing a novel sub-clade of the thalassiosiroid diatoms; however, furthermore culture based study can confirm the same. We cannot rule out the other possibility that it could be because of the limited number of *rbc*L sequences currently available in published databases. Five *rbc*L sequences in clade1 (three from StnSBRN1, one from StnSBRN2 and one from StnSBRS1) clustered with *T. sundarbana*. Incidentally, *T*. *sundarbana* is a new species of *Thalassiosira* that has been recently discovered and described from an estuarine environment in Sundarbans (Samanta and Bhadury, 2015). The detection of *rbc*L signatures of *T. sundarbana* additionally from SBR suggested that this species may be indigenous to Sundarbans mangrove and also has a broad distribution throughout the ecosystem.

In clade2, a total of 24 clones clustered with the species of two asymmetrical bi-raphid pennate diatom genera *Amphora* and *Halamphora* (Fig. S1). The *rbc*L clones in this clade showed highest identity (98–99%) with *Amphora caribaea* and *Halamphora montana*. In clade3, 23 clones clustered with a pennate diatom *Bacillaria paxillifer*, however, showed only 95–96% identity with this species (Fig. S1). The statistical support of the node separating *B. paxillifer* from our *rbc*L clone cluster is also very weak both in ML (bootstrap value <50%) and Bayesian (posterior probability value <0.95) analyses. In addition to these three major clades, one *rbc*L clone from StnSBRN2 clustered with *rbc*L sequence of *Nitzschia filiformis*, one clone from StnSBRS1 clustered with *Petrodictyon gemma* and another clone from StnSBRN2 clustered with *Chaetoceros dayaensis* (Fig. S2).

In the present study, several new records of diatom species from the Indian part of SBR were reported using light microscopy and several novel *rbc*L sequences were detected in the phylogeny which represents an important set of baseline information for diatom assemblages from a mangrove ecosystem. One of the constraints of this study is that the microscopic observation and the molecular identification of the diatoms had been undertaken independently from each environmental sample. This could be one of the reasons for the lack of correlation between morphological and molecular data. However, our results from *rbc*L clone library approach provide first data on the diversity of diatom species in the study area and also highlight the need to expand published sequence databases by adding *rbc*L sequences representing unique ecosystems such as mangroves. For DNA barcoding of diatoms, Hamsher et al. (2011) showed that *rbc*L gene could be good selection based on its variance, whereas Zimmermann and colleagues affirmed that V4 region of SSU rDNA could be a good alternative for diatom barcoding (Zimmermann et al., 2011, 2014, 2015). The 5.8S+ITS2 could also be another alternative barcoding marker for diatoms (Moniz and Kaczmarska, 2010). Our present study suggests that it is important to establish the cultures for better understanding of diatom assemblages in the mangrove waters. To create an authenticated diatom database from the mangrove waters, it is also important to characterize those established cultures using multigene phylogeny and detailed morphological documentations using electron microscopy.

## 4 Conclusions

Overall this study has documented several new records of diatom species from Indian part of SBR using microscopy, which has hitherto remained unknown so far. As part of this study, the *rbc*L clone library approach provides glimpse of diversity of diatoms from the study area and also highlight that the published sequence databases needs to be populated further with *rbc*L sequences representing unique ecosystems such as mangrove ecosystems. With respect to future ecosystem monitoring of SBR, reported diatom species along with *rbc*L phylogeny provide baseline information of diatom assemblages. This study indicates that establishment of cultures and their polyphasic taxonomy are essential to create an authenticated diatom database from mangrove water, which is still overlooked globally.

## Declarations

### Author contribution statement

Brajogopal Samanta: Conceived and designed the experiments; Performed the experiments; Analyzed and interpreted the data; Wrote the paper.

Punyasloke Bhadury: Conceived and designed the experiments; Contributed reagents, materials, analysis tools or data; Wrote the paper.

### Funding statement

This work is partly supported by MoES grant (MMME of MLRP through CMLRE) and start up grant of IISERK awarded to Punyasloke Bhadury. Brajogopal Samanta is the recipient of an IISERK PhD Fellowship.

### Competing interest statement

The authors declare no conflict of interest.

### Additional information

Data associated with this study has been deposited at Genbank under the accession numbers KT335295-304, KT335317-324, and KT335346-398.

## Figures and Tables

**FigureS1:**
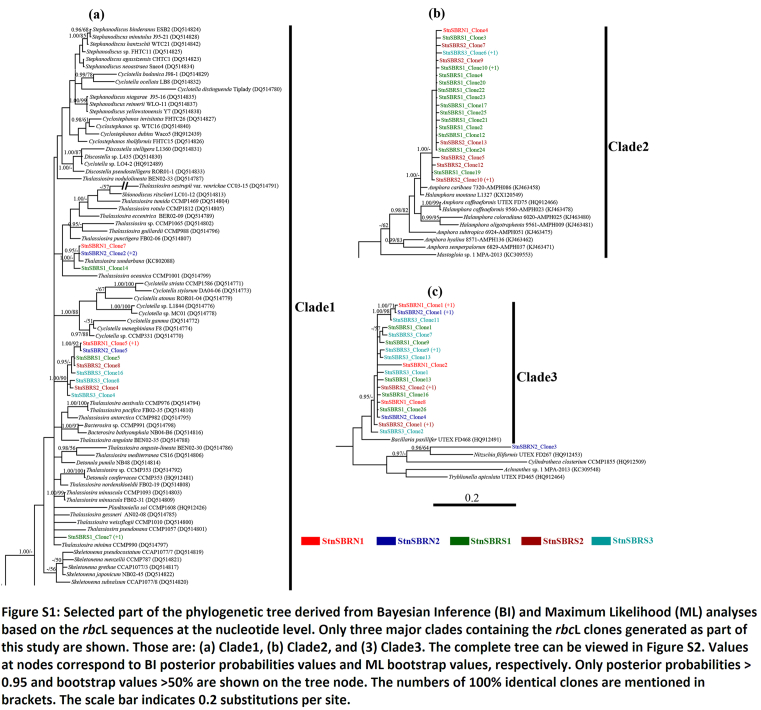
Selected part of the phylogenetic tree derived from Bayesian Inference (BI) and Maximum Likelihood (ML) analyses based on *rbc*L sequences at the nucleotide level. Only three major clades containing the *rbc*L clones generated as part of this study are shown. Those are: (a) Clade1, (b) Clade2, and (c) Clade3. The complete tree can be viewed in Supplementary Fig. S2. Values at nodes correspond to BI posterior probabilities values and ML bootstrap values, respectively. Only posterior probabilities >0.95 and bootstrap values >50% are shown on the tree node. The numbers of 100% identical clones are mentioned in brackets. The scale bar indicates 0.2 substitutions per site.
